# Risk and protective factors of health-related quality of life in children and adolescents: Results of the longitudinal BELLA study

**DOI:** 10.1371/journal.pone.0190363

**Published:** 2017-12-28

**Authors:** Christiane Otto, Anne-Catherine Haller, Fionna Klasen, Heike Hölling, Monika Bullinger, Ulrike Ravens-Sieberer, on behalf of the BELLA study group

**Affiliations:** 1 Department of Child and Adolescent Psychiatry, Psychotherapy, and Psychosomatics, University Medical Center Hamburg-Eppendorf, Hamburg, Germany; 2 Department of Epidemiology and Health Monitoring, Robert Koch-Institute, Berlin, Germany; 3 Institute and Outpatients Clinic Medical Psychology, Center for Psychosocial Medicine, University Medical Center Hamburg-Eppendorf, Hamburg, Germany; Chiba Daigaku, JAPAN

## Abstract

**Aims:**

Cross-sectional studies demonstrated associations of several sociodemographic and psychosocial factors with generic health-related quality of life (HRQoL) in children and adolescents. However, little is known about factors affecting the change in child and adolescent HRQoL over time. This study investigates potential psychosocial risk and protective factors of child and adolescent HRQoL based on longitudinal data of a German population-based study.

**Methods:**

Data from the BELLA study gathered at three measurement points (baseline, 1-year and 2-year follow-ups) were investigated in *n* = 1,554 children and adolescents aged 11 to 17 years at baseline. Self-reported HRQoL was assessed by the KIDSCREEN-10 Index. We examined effects of sociodemographic factors, mental health problems, parental mental health problems, as well as potential personal, familial, and social protective factors on child and adolescent HRQoL at baseline as well as over time using longitudinal growth modeling.

**Results:**

At baseline, girls reported lower HRQoL than boys, especially in older participants; low socioeconomic status and migration background were both associated with low HRQoL. Mental health problems as well as parental mental health problems were negatively, self-efficacy, family climate, and social support were positively associated with initial HRQoL. Longitudinal analyses revealed less increase of HRQoL in girls than boys, especially in younger participants. Changes in mental health problems were negatively, changes in self-efficacy and social support were positively associated with the change in HRQoL over time. No effects were found for changes in parental mental health problems or in family climate on changes in HRQoL. Moderating effects for self-efficacy, family climate or social support on the relationships between the investigated risk factors and HRQoL were not found.

**Conclusion:**

The risk factor mental health problems negatively and the resource factors self-efficacy and social support positively affect the development of HRQoL in young people, and should be considered in prevention programs.

## Introduction

Health-related quality of life (HRQoL) is a subjective and multidimensional construct that includes physiological, psychological, and functional aspects of health and wellbeing [1]. Generic HRQoL in children and adolescents includes dimensions specifically related to the children’s and the adolescents’ experience and environment, such as physical and psychological wellbeing, family life, school environment, and peer relations [2]. HRQoL has become a major health outcome in epidemiological research on children and adolescents [3, 4]. Identifying the factors that affect child and adolescent HRQoL can provide important information for policy makers, can help to develop effective prevention programs for children and adolescents [5], and will thereby promote and enable positive mental and physical health and well-being in the long term [6, 7].

A number of standardized age-appropriate measures are available to measure HRQoL in children and adolescents [8, 9]. HRQoL is a subjective perception, it is therefore best assessed by means of self-reports [10, 11], as long as the measures are age-appropriate [9, 10, 12]. Parent- and proxy-reports may be gathered in addition, or if the children are very young or suffer from very severe health conditions [8, 9, 11].

In recent years, research in public health has increasingly been influenced by the concept of risk and protective factors and their effects on mental health and well-being [13–15]. Whereas risk factors increase the probability of occurrence of a negative health outcome, protective factors in contrary decrease the probability of undesirable outcomes or increase the probability of positive development of the outcome of interest [16]. Within this theoretical framework, more complex interactions are possible, with protective factors for instance moderating the detrimental effect of risk factors on the outcome of interest [16–18]. To our knowledge, the concept of risk and protective factors has to date only been applied to child and adolescent HRQoL in studies on clinical samples using cross-sectional data (e.g., in children with chronic health conditions [19]).

Sociodemographic factors associated with child and adolescent HRQoL have been investigated in numerous population- or school-based cross-sectional studies. Analyses from data of large surveys such as the European KIDSCREEN study (*n* = 21,590) show that general HRQoL was higher in younger compared with older participants [20]. Furthermore, with increasing age, girls reported lower HRQoL than boys; this gender-specific difference was more pronounced in older compared with younger children and adolescents in the investigated sample [20]. In a population-based study conducted in seven European countries, higher parental education, better work status, and higher family wealth were associated with higher HRQoL in children and adolescents (*n* = 1,896 aged 8 to 18 years) [21]. Similarly, findings of the international study on Health Behavior of School-aged Children (HBSC study) based on European data from 15 countries (*n* = 78,000 pupils aged 11, 13, and 15 years) showed that the family’s socioeconomic status was positively associated with the pupils’ HRQoL [22].

Concerning associations of mental health and HRQoL in children and adolescents, research based on a large European cross-sectional, population-based sample of almost 16,000 children and adolescents showed that child mental health problems were associated with lower HRQoL [23]. In line with these findings, Sharpe et al. [24], using data from a school-based study surveying 45,398 pupils aged 8 to 13 years in England, reported a strong link between mental health problems and HRQoL (group comparisons showed the highest HRQoL score for children with no mental health problems, and the lowest HRQoL score for those with both internalizing as well as externalizing mental health problems). Yet, the authors also found that 12% of the investigated children with mental health problems reported high HRQoL [24]. Further, parent-reported parental mental health problems have also been shown to be associated with lower HRQoL children and adolescents based on data of a Greek study (*n* = 1,194 children and adolescents aged 11 to 17 years) [25]. These findings are in line with a model on determinants of child and adolescent HRQoL developed by the KIDSCREEN group suggesting that HRQoL is associated not only with sociodemographic factors, but additionally with physical and mental health of the children and adolescents, with parental mental health, as well as with parent-child relationships [8].

Concerning psychosocial factors associated with child and adolescent HRQoL, the extensive body of literature on social support compiled over several decades has recognized the major influence social support has on mental health and well-being [26, 27]. Moreover, analyses based on baseline data of the German BELLA study of children and adolescents aged 7 to 17 years (*N* = 2,863) revealed that personal (i.e., self-efficacy, optimism, sense of coherence), familial (i.e., positive family climate), and social (i.e., social support) factors explained more than 50% of the variance of HRQoL [28]. Kvarme et al. [29] found positive associations between self-efficacy and HRQoL in a sample of 279 school children from 7^th^ grade. A cross-sectional study using data of over 22,830 children and adolescents from 13 European countries found positive associations between parent-child relationship and social support and HRQoL [28]. Similarly, in their national sample of 3,195 Portuguese 5^th^ and 7^th^ grade pupils Gaspar et al. [30] found that self-esteem, optimism, and social support had a considerable positive impact on the pupils’ HRQoL.

As far as we know, there has only been little research on the effects of factors on individual changes in child and adolescent HRQoL over time that go beyond sociodemographic data and include psychosocial factors. Based on longitudinal data of the population-based German BELLA study, Barkmann et al. [31] investigated five domains of HRQoL as measured by means of the KIDSCREEN-27 [8]. Barkmann et al. demonstrated that older age, female gender, lower socioeconomic status, and migration background were predictors of lower HRQoL in children and adolescents (*n* = 1,597 aged 11 to 17 years) [31]. The authors furthermore suggest that the age and gender differences in HRQoL may reflect hidden processes associated to puberty, the changes, and the developmental challenges related to this particular developmental period [31]. A negative association of a migration background and HRQoL was also found in a trend analysis using data of more than 10,000 Dutch children aged 4 to 11 years [5]. However, to our knowledge, there is no study that has looked at psychosocial risk as well as protective predictors of child and adolescent HRQoL based on longitudinal data.

The aims of this study are to examine the effects of selected psychosocial risk and protective factors on child and adolescent overall HRQoL in a German population-based sample of the longitudinal BELLA study initially (at baseline) as well as over a period of two years. We focused on overall HRQoL and controlled for effects of sociodemographic factors investigating effects of potential risk factors (i.e., mental health problems and parental mental health problems) as well as protective factors (i.e., self-efficacy, positive family climate, and social support) on overall HRQoL cross-sectionally as well as longitudinally using self-reported data. We expected that mental health problems and parental mental health problems were negatively, and self-efficacy, positive family climate as well as social support were positively associated with initial HRQoL and with the change in HRQoL over time. Moreover, we expected that self-efficacy, family climate, and social support attenuate the relationship between the investigated risk factors (i.e., mental health problems and parental mental health problems) and HRQoL initially as well as over time.

## Methods

### Study design

The longitudinal BELLA study is the mental health module of the German National Health Interview and Examination Survey among Children and Adolescents (KiGGS study). The BELLA study gathers data on mental health and well-being of children and adolescents. The baseline assessment of the BELLA study took place between 2003 and 2006 with a number of 2,863 children and adolescents (aged 7 to 17 years) participating. Further measurement points were conducted in the BELLA study including a 1-year (2004 to 2007) and a 2-year follow-up (2005 to 2008). Data were collected by means of telephone interviews and subsequent questionnaires. Self-reported information of children and adolescents were gathered if participants were aged at least 11 years. Additionally, parent-reported data were gathered from one parent of each participant. Written informed consent was obtained from adolescents (aged at least 14 years) and from parents (for themselves as well as for their participating children younger than 14 years) at each measurement point. Approval for the BELLA study was obtained from the ethics committee of the Federal Commissioner for Data Protection in Germany. The BELLA study was performed in accordance with the ethical standards laid down in the 1964 Declaration of Helsinki and its later amendments. More details on the conceptualization, design, and methods of the BELLA study as well as the KiGGS study have been published [32, 33].

### Participants

In the present study, we analyzed data from the first three measurement points of the BELLA study gathered over a period of two years (at baseline, 1-year, and 2-year follow-ups). To allow the inclusion of a participant in our analyses, data on age, gender, the socioeconomic status (SES), and migration background had to be available; further, valid data for at least one of the three investigated measurements points were necessary regarding HRQoL, mental health problems, parental mental health problems, self-efficacy, family climate, as well as social support. The final sample under analysis included 1,554 children and adolescents aged 11 to 17 years at baseline who had participated in at least one of the three consecutive measurement points of the BELLA study.

### Measures

#### Sociodemographic variables

Age, gender, SES, and migration background were assessed at baseline. The SES was measured using the Winkler Index, a score that integrates information regarding the total household income as well as parental educational and occupational status [34] into one metric variable ranging from 3 to 21. In order to differentiate between participants, and only for sample description, the score of the Winkler-Index was categorized into groups with low (3 to 8 points), medium (9 to 14 points), and high SES (15 to 21 points) [35]. Further, the potential migration background of participants was assessed based on Schenk [36]. Migration background was assumed if i) the child or adolescent had immigrated to Germany and had at least one parent foreign born or if ii) both parents of the participant immigrated to Germany or did not hold German citizenship.

#### Health-related quality of life

The self-report version of the KIDSCREEN-10 Index [8] was administered at each investigated measurement point of the BELLA study. The KIDSCREEN-10 Index provides a global HRQoL score covering physical, psychological, and social facets of HRQoL. It has good discriminatory power and its strong internal consistency (Cronbach’s α = .82) as well as its test-retest reliability (Intra Class Correlation (*ICC*) = .70) as found in a large international sample allow precise and stable measurements [37]. The KIDSCREEN-10 Index includes ten items (e.g., “Have you felt fit and well?” or “Have you had enough time for yourself?”) offered with 5-point response scales (0 = *not at all* to 4 = *extremely* or 0 = *never* to 4 = *always)*. For scoring, we recoded negatively phrased items and calculated the index score across all ten items as a mean score ranging from 0 to 4. Good internal consistency for the KIDSCREEN-10 Index was found in the present study (Cronbach’s α ranged from .78 to .82 across the investigated measurement points). Solely for sample description, we calculated a T-score for the KIDSCREEN-10 Index based on European reference data [8]; by means of these T-scores (Mean (*M*) = 50; Standard Deviation (*SD*) = 10) participants were categorized into three groups with low (T-scores < 40), medium (T-scores ranging from 40 to 60) and high HRQoL (T-scores > 60) [38].

#### Mental health problems

Mental health problems (of children and adolescents) were assessed using the German self-report version of the Strengths and Difficulties Questionnaire (SDQ) [39] at each measurement point of the present study. The SDQ is a well-established, reliable, and valid measure to assess emotional and behavioral problems in the past six months. It includes four problem scales on emotional symptoms, conduct problems, hyperactivity/inattention, and peer problems consisting of five items each. SDQ items are offered with three response options (0 = *not true*, 1 = *somewhat true*, 2 = *certainly true*). The Total Difficulties Score of the SDQ can be calculated summarizing all 20 items of the four problem scales. In the present study, a mean was calculated for the Total Difficulties Score ranging from 0 to 2. Good internal consistency for the SDQ Total Difficulties Score was detected across the measurement points in the investigated sample (Cronbach’s α ranged from .72 to .75). For descriptive purposes only we created two groups based on the Total Difficulties score differentiating children and adolescents with mental health problems (gathering SDQ categories *borderline* and *abnormal*) from those without mental health problems (SDQ category *normal)* using a published cut-off [40].

#### Parental mental health problems

The short form of the German version of the Symptom Checklist-90-R (SCL-90-R) [41], i.e., the SCL-S-9 [42] was administered to the participating parents at each measurement point of the study. By means of nine items, the SCL-S-9 measures mental health problems in the past seven days with regard to depression, anxiety, somatization, obsessive-compulsive symptoms, anger-hostility, interpersonal sensitivity, phobic anxiety, paranoid ideation, and psychoticism. The items of the SCL-S-9 are offered with a 5-point response scale (0 = *not at all* to 4 = *very much*). In the BELLA study, we gathered parent-reported information of one parent for each participating child or adolescent; the decision which parent participated was primarily based on availability or willingness. For 89% of the cases, the biological mother filled in the questionnaire, followed by the biological father with 10% of the cases; for the remaining 1% cases, the SCL-S-9 was filled in by other care-givers such as stepparents, grandparents or adoptive or fostering parents. For the analyses, we calculated a mean across all nine items of the SCL-S-9 ranging from 0 to 4 with a higher mean indicating more severe parental mental health problems. Good internal consistency for the SCL-S-9 was found in the present study (Cronbach’s α ranged from .81 to .84 across the measurement points). Only for the purpose of sample description, we categorized the children and adolescents into two groups of participants with parents with and without mental health problems based on the SCL-S-9 score (using a recommended cut-off of the mean plus two standard deviations of a reference sample or a higher score as indicator for given mental health problems [42]).

#### Self-efficacy

The General Self-Efficacy Scale (SE) [43, 44] was used to assess self-reports of children and adolescents at each measurement point of the study. The ten items of the SE (e.g., “It is easy for me to stick to my aims and accomplish my goals”) are rated on a 4-point-scale (0 = *not at all true* to 3 = *exactly true*). In the present study, a mean across all ten items was calculated ranging from 0 to 3 with a higher mean indicating higher self-efficacy. The internal consistency of the SE in the investigated sample was good (Cronbach’s α ranged from .80 to .83 across measurement points).

#### Family climate

Family climate was measured by children’s and adolescents’ self-reports using eight items of the German Family Climate Scale (FCS) [45] at each measurement point of the study. The FCS is the German adaptation of the Family Environment Scale (FES) [46]. The administered eight items of the FCS pertain to the domains family cohesion (e.g., “In our family everybody cares about each other’s worries”) as well as recreational activities (“We often go to the cinema, visit sport events or go on excursions”), and are offered with a 4-point response scale (from 0 = *not true* to 3 = *exactly true*). We recoded negatively phrased items and calculated a mean score over the administered eight items of the FCS that ranged from 0 to 3 with higher values indicating better family climate. Good internal consistency was found for the investigated 8-item FCS score in the investigated sample (Cronbach’s α ranged from .78 to .83 across measurement points).

#### Social support

Social support was measured based on the Social Support Survey (SSS) [47] at each measurement point of the study. We used a self-report short version of the German translation of the SSS which is applicable to children and adolescents (SSS-short). The eight items of the administered SSS-short assess how often specific types of support were available for the child or adolescent (e.g. “How often is the following type of support available for you if you need it?: Someone you can count on to listen to you, when you need to talk”) by means of a 5-point rating-scale (0 = *none of the time* to 4 = *all of the time*). The calculated mean over all items of the SSS-short ranged from 0 to 4 with a higher mean representing more available social support. Good to excellent internal consistency was found for the SSS-short in the present study (Cronbach’s α ranged from .88 to .91).

### Data analyses

We used a two-step approach to investigate the effects of the above described risk and protective factors on HRQoL initially as well as over time. At first, we calculated a latent growth model (LGM) for each construct included in our analyses (i.e., HRQoL, mental health problems, parental mental health problems, self-efficacy, family climate, and social support). LGMs are frequently used in social, psychological, and health research for the analysis of longitudinal data. This method is particularly suitable for analyses of change in behavior [48]. By means of LGMs, two latent factors, i.e., intercept and slope, can be estimated using a regression-type line of the variable of interest over time. The intercept represents the initial status of the variable at baseline and the slope shows the change in the same variable over time. In the present study, we used the calculated intercepts and slopes for each construct in subsequent linear regression analyses. Regression Model A0 served to investigate whether initial HRQoL was predicted by initial mental health problems, parental mental health problems, self-efficacy, family climate, and social support (using only intercepts). Regression Model B0 was used to analyze whether the change in HRQoL (slope) was predicted by the initial states of mental health problems, parental mental health problems, self-efficacy, family climate, and social support (intercepts), as well as by the change in these variables over time (slopes). Age, gender, SES, and migration background were included in both regression models as covariates; further, we added the interaction of age by gender to both models. Prior to conducting regression analyses, we centered metric predictors to be included in Models A and B.

By means of further regression models we investigated whether the relationships between HRQoL and the examined risk factors were moderated by the investigated protective factors. Model A1 served to investigate potential moderating effects of initial protective factors self-efficacy, family climate, and social support on the association between initial mental health problems and initial HRQoL. Correspondingly, Model A2 was used to analyze whether the initial protective factors self-efficacy, family climate, and social support moderated the relationship between initial parental mental health problems and initial HRQoL. Subsequently, we analyzed whether associations between the change in HRQoL and the initial state of mental health problems as well as the change in mental health problems were moderated by self-efficacy, family climate, and social support using Model B1. Finally, we investigated whether associations between the change in HRQoL and the initial state of as well as the change in parental mental health problems were moderated by self-efficacy, family climate, and social support with Model B2. We controlled for effects of sociodemographic variables (i.e., age, gender, SES, migration status, and the interaction between age and gender) in all moderator models.

To evaluate the strengths of the detected effects based on standardized regression weights, we used Cohen’s rules of thumb [49] with β = .1 indicating a small, β = .3 a medium, and β = .5 a strong effect. The LGMs were calculated using Mplus 6.11 [50], regression analyses were conducted using IBM SPSS 22.

## Results

The analyzed sample of children and adolescents aged 11 to 17 years at baseline (*n* = 1,554) is described in Table 1. About half of the investigated children and adolescents were female (51%), the mean age was 13.9 years (*SD* = 2.97), and 8% of the children and adolescents had a migration background. Moreover, a proportion of 23% of the participants had low, 51% medium, and 27% had high SES. Further, a proportion of 11% of the children and adolescents reported mental health problems (according to SDQ groups *borderline* and *abnormal*) and 8% of the participants had parents with mental health problems at baseline. Self-reported HRQoL was low in 8% of the participants, moderate for 77%, and high in 15% of the participants at baseline.

**Table 1:** Description of the analyzed sample of children and adolescents aged 11 to 17 years (at baseline).

	Baseline		1-year follow-up		2-year follow-up	
	*n (%)*	*M* (*SD*)	*n*	*M* (*SD*)	*n*	*M* (*SD*)
**Sociodemographic data**^1^						
Female	785 (51%)					
Age (in years)		13.90 (1.97)				
Socioeconomic status		11.81 (4.16)				
Migration background	126 (8%)					
**Risk factors**						
Mental health problems	1,538	0.49 (0.23)	1,213	0.40 (0.22)	1,185	0.38 (0.22)
Parental mental health problems	1,536	0.60 (0.52)	1,241	0.60 (5.34)	1,212	0.51 (0.51)
**Protective factors**						
Self-efficacy	1,527	2.13 (0.38)	1,210	2.15 (0.44)	1,184	2.17 (0.41)
Family climate	1,538	1.83 (0.53)	1,171	1.83 (0.52)	988	1.80 (0.53)
Social support	1,533	3.12 (0.74)	1,175	3.29 (0.67)	992	3.32 (0.65)
**Health related quality of life**	1,378	3.09 (0.53)	1,199	3.20 (0.51)	1,138	3.18 (0.53)

^1^ Sociodemographic data were available for the total sample under analysis (*n* = 1,554).

*M* = mean, *SD* = standard deviation; for measures, see text (Methods).

We calculated LGMs including a linear growth for each investigated construct (i.e., HRQoL, mental health problems, parental mental health problems, self-efficacy, family climate, and social support). Intercepts and slopes resulting from LGMs were used for the subsequent regression analyses.

Results for regression Model A0 (Table 2) showed that female gender, older age, lower SES, and migration background were all associated with lower HRQoL at baseline. The significant interaction effect indicated that the gender-specific difference in HRQoL was more pronounced in older compared to younger children and adolescents. Further, the potential risk factors mental health problems as well as parental mental health problems were both negatively associated with initial HRQoL. Moreover, the potential protective factors self-efficacy, family climate, and social support were all positively related to HRQoL at baseline. The standardized regression coefficients showed that mental health problems had a medium effect on HRQoL in children and adolescents, whereas for parental mental health problems only a very small to small corresponding effect was found. For protective factors, consistently small effects on initial HRQoL were found.

**Table 2:** Predictors of the initial status and the change in child and adolescent health-related quality of life.

	Regression Model A0^1^ predicting initial HRQoL			Regression Model B0^2^ predicting change in HRQoL		
	*b*	β	*p*	*b*	β	*p*
*Constant*	*3*.*12*		< .*001*	*0*.*05*		< .*001*
**Sociodemographic data**						
Female	-0.10	-.14	< .001	-0.01	-.06	.012
Age (in years at baseline)	-0.03	-.17	< .001	0.00	.03	.443
Age by gender	-0.01	-.06	.031	0.00	.09	.007
Socioeconomic status (at baseline)	-0.01	-.07	< .001	0.00	.04	.132
Migration background	-0.05	-.04	.026	-0.01	-.04	.095
**Risk factors**						
Initial mental health problems (intercept)	-0.74	-.34	< .001	-0.00	-.01	.719
Initial parental mental health problems (intercept)	-0.07	-.08	< .001	0.00	.02	.389
Change in mental health problems (slope)				-0.28	-.29	< .001
Change in parental mental health problems (slope)				-0.03	-.03	.207
**Protective factors**						
Initial self-efficacy (intercept)	0.23	.17	< .001	-0.01	-.03	.291
Initial family climate (intercept)	0.15	.16	< .001	-0.01	-.05	.099
Initial social support (intercept)	0.13	.16	< .001	0.00	.03	.282
Change in self-efficacy (slope)				0.07	.15	< .001
Change in family climate (slope)				0.02	.04	.094
Change in social support (slope)				0.04	.09	.001

^1^ Linear regression Model A0 (*n* = 1,554); model fit: adjusted *R*^*2*^ = .49; *F* = 151.22.

^2^ Linear regression Model B0 (*n* = 1,554); model fit: adjusted *R*^*2*^ = .16; *F* = 20.82.

HRQoL = health-related quality of life; *b* = unstandardized regression coefficient; β = standardized regression coefficient; for measures see text (Methods).

**Fig 1:**
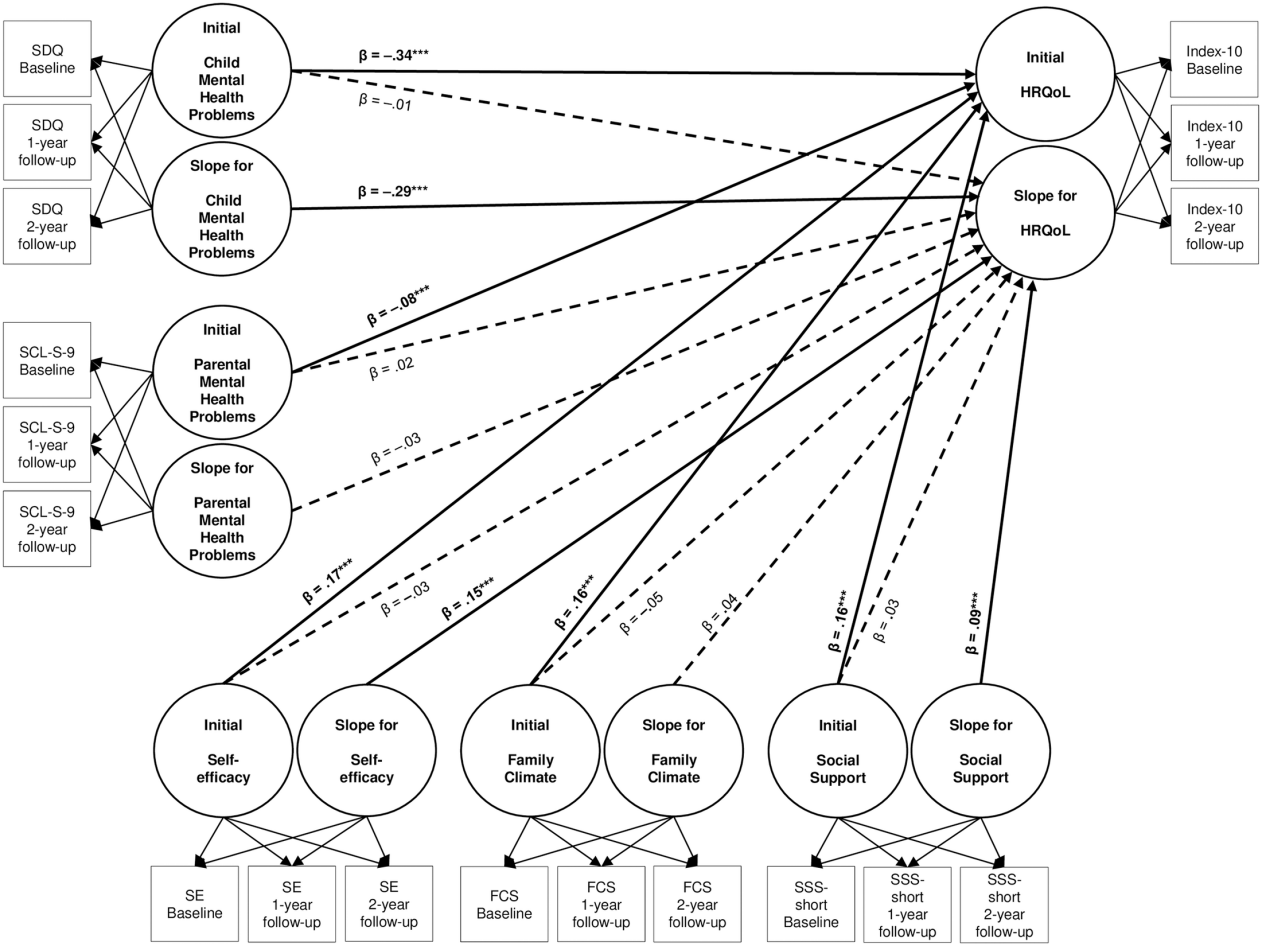
Effects of risk and protective factors on health-related quality of life in children and adolescents. Continuous lines mark significant effects, interrupted lines indicate non-significant effects, resulting from regression Models A and B. *n* = 1,554. HRQoL = Health-related quality of life measured with the KIDSCREEN-10 Index [8]; SDQ = Strengths and Difficulties Questionnaire [39]; SCL-S-9 = Symptom-Check List Short version-9 [41, 42]; SE = General Self-Efficacy Scale [43,44]; FCS = an eight-item score based on the Family Climate Scale [45,46]; SSS-short = eight items of the German version of the Social Support Survey [47]; β = standardized regression coefficient; ***p ≤ .001.

The findings for regression Model B0 (Table 2) showed less increase in HRQoL over time for girls compared to boys; the significant interaction effect indicated that this gender-specific difference in the change in HRQoL was more pronounced in younger compared to older children and adolescents. Concerning potential risk and protective factors, we found no effects of the initial states in these factors on the change in HRQoL over time. However, increasing mental health problems were associated with decreasing HRQoL in children and adolescents over time. No effect was found for the change in parental mental health problems on the change in child and adolescent HRQoL. Concerning potential protective factors, improving self-efficacy as well as improving social support were both associated with an improvement in HRQoL over time. No significant association between the changes in the family climate and in HRQoL over time could be found. According to Cohen [49], the effect of the change in mental health problems on the change in HRQoL was small to medium, the effect of the change in self-efficacy was small, and the effect for the change in social support was very small to small. The results for both regression models are integrated in Fig 1.

Further regression models served to investigate potential moderating effects of the investigated protective factors on the relationships between the examined risk factors and HRQOL. However, the results indicated no moderating effect for any protective factor on the relationships of HRQoL with mental health problems (Models A1 and B1) or with parental mental health problems (Models A2 and B2). The corresponding results are presented as Supporting Information (Tables A and B in S1 File).

## Discussion

The aims of the present study were to provide population-based findings concerning the effects of potential risk and protective factors on overall child and adolescent HRQoL initially (at baseline) as well as over time. Besides effects of the sociodemographic variables age, gender, SES, and migration background we found negative effects of the investigated risk factors (i.e., mental health problems and parental mental health problems), and positive effects of the investigated personal, familial, and social protective factors (i.e., self-efficacy, family climate, and social support) on the HRQoL in children and adolescents at baseline. In our longitudinal analysis, we detected an interaction effect of age by gender as well as a negative effect of the change in the risk factor mental health problems, and positive effects of changes in the protective factors self-efficacy and social support on the change in HRQoL over the course of two years. In more detailed analyses, we detected no moderating effects of any protective factor on the relationships between HRQoL and the investigated risk factors.

Our analyses based on baseline data indicated that female gender and older age were negatively associated with initial HRQoL, with the gender-specific difference being more pronounced in older compared to younger participants. These findings are in line with results from previous cross-sectional population- and school-based studies [20, 51]. We further found a negative effect of SES on initial HRQoL. However, former studies in general population or school-based samples reported positive associations between socioeconomic variables and HRQoL in children and adolescents [21, 22]. Yet, the negative effect in our analyses of SES on initial HRQoL was very small (β = —.07) and more detailed a-posteriori investigations revealed this to be a suppressor effect (SES did not correlate with initial HRQoL (*r* = .01; *p* = .600), nor could we detect a substantial correlation for SES with any other construct included in our analyses). Moreover, a given migration background was associated with lower initial HRQoL in our sample, thereby confirming results from previous studies investigating this relationship [31, 5].

Regarding the associations of risk and protective factors with HRQoL at baseline our analyses revealed expected results: In line with the existing body of literature [23, 25], the investigated risk factors mental health problems and parental mental health problems had negative effects on initial HRQoL. Further, positive associations were found between the investigated protective factors (i.e., self-efficacy, positive family climate, and social support) with initial HRQoL, thereby corroborating results from previous cross-sectional studies based on population- or school-based samples [28–30].

Our longitudinal analyses indicated that the increase over time of girls’ HRQoL was less pronounced compared with the increase of boys’ HRQoL. This finding may reflect the fact that the challenging transition period between childhood and adulthood affects girls differently compared to boys (for a detailed description of changes and processes involved see e.g., [52–54]). We further detected that the gender-specific difference in the change of HRQoL was more pronounced in younger compared to older children and adolescents. This effect may be due to the earlier onset of puberty in girls compared with boys [55] and the related changes in hormonal states that have been shown to be associated with changes of mood and behavior—particularly in younger girls [54, 56]; which in turn may also affect HRQoL and well-being. Furthermore, the bodily changes associated with the onset of puberty—which are more visible in girls compared with boys—may give rise to actual or perceived social reactions, leading to decreased mental health and well-being in girls compared with boys [52, 57].

In our longitudinal analyses we moreover found that change in mental health of the children and adolescents was associated with change in HRQoL. Also, changes in self-efficacy and social support were related to change in HRQoL. These findings based on longitudinal data are in line with results from former studies based on cross-sectional data and underline the importance to consider mental health as a risk factor and self-efficacy as well as social support as important protective factors of HRQoL in children and adolescents. Longitudinal studies on the development of HRQoL in children and adolescents are scarce. The majority of the existing studies investigated the effects of age and gender on HRQoL over a period of six months [58–60] or of up to three years [61–63]. None of these studies investigated the risk and protective factors examined in our study. Barkmann et al. [31] however investigated a comprehensive model on the effects of several factors including child and parental mental health problems on the five domains of HRQoL according to the KIDSCREEN-27 (i.e., physical well-being, psychological well-being, relationship with parents & autonomy, peers & social support, and school environment) over the course of two years based on the same data set as analyzed in the present study. In comparison, we investigated an overall HRQoL score according to the KIDSCREEN-10 Index, focused on risk and protective factors, and chose a different method of analysis compared to those authors. Overall, the results of both studies underline the importance of considering the risk factor mental health problems for the development of HRQoL in children and adolescents.

For our longitudinal model the proportion of the explained variance in the outcome was only 16% (compared to 49% for our baseline model). We found a small to medium effect for the change in mental health problems on the change in HRQoL, but the remaining effects were only very small to small, if given at all. We believe that our longitudinal results at least partly reflect the fact that we investigated a general population sample with a consistent level of rather good HRQoL, little mental health problems, as well as with good self-efficacy, family climate, and social support; moreover, our study only covered a period of two years. These aspects are most apparent regarding the slope for parental mental health problems, which did not vary substantially across individuals. However, our results may also indicate that the development of HRQoL over time may be associated with factors that we did not consider in our model. These factors may include health-related variables such as physical health and chronic disorders of the children and adolescents [64, 65], and further potential predictors such as variables related to adolescence (e.g., body image, peer relationships, and bullying [57], pubertal development [62]), or academic demands at school [28].

Our results indicate that the investigated factors may have direct effects on HRQoL, but we found no evidence for moderating effects of self-efficacy, family climate or social support on the relationships between the risk factors and HRQoL by means of our moderator models. Following the distinction between protective and resource factors as summarized and suggested by Rose et al. [66], self-efficacy, family climate and social support should be described as resource factors of child and adolescent HRQoL based on our findings. Similarly, Otto et al. [67] did not find any moderating effects of personal, familial and social factors on the investigated relationship between the risk factor parental anxiety and child generalized anxiety in their study based on the same longitudinal data set of the BELLA study. However, for depressive symptoms in children and adolescents, Klasen et al. [68] reported moderator effects of familial and social protective factors (i.e., family climate and social support) on the relationship between parental mental health problems and child depressive symptoms using longitudinal data from the German BELLA study. Future research focusing on risk and protective or resource factors of HRQoL may wish to further analyze other potential kinds of relationships of such factors with HRQoL (compare [16–18]).

The German BELLA study is a large population-based cohort study on child and adolescent well-being and mental health. Numerous studies based on data from the BELLA study have been published including studies on HRQoL (e.g., [69,31]) as well as studies on the prevalence and trajectories of mental health problems in children and adolescents [70–72]. Moreover, in a recently published cross-sectional analysis based on data from the 6-year-follow-up of the population-based BELLA study, risk factors for parental as well as for child mental health problems were investigated [73]. In our present study, we focused on HRQoL and analyzed longitudinal data of the BELLA study. The present study adds to the body of literature findings on factors affecting child and adolescent overall generic HRQoL initially as well as over time. Our results may help to inform policy makers when planning health literacy and health promotion programs. In particular, we identified modifiable factors related to child and adolescent health and well-being [4] that may also be of interest with regard to empowerment of children and adolescents [74, 75].

This study has the following limitations. Our longitudinal data only covered a period of two years; future studies may wish to analyze the development of HRQoL in children and adolescents over a longer period of time. Moreover, future studies may wish to investigate effects of further risk and protective or resource factors on HRQoL, which were not included in the analyses of the present study. The present study has the following strengths: We investigated data from the BELLA study, an important cohort study on the well-being and mental health of children and adolescents in Germany administering established measurement tools. Further, we used an appropriate and modern method of analysis that allowed us to examine our metric data without any loss of information compared to methods analyzing categorized data. However, future studies may wish to use another method such as latent class modeling in order to identify groups based on longitudinal HRQoL data and subsequently may wish to investigate these groups in greater detail.

Overall, the present study shows how important children’s and adolescents’ mental health is with regard to their HRQoL. Furthermore, we were able to demonstrate the beneficial effects of self-efficacy and social support on HRQoL in children and adolescents over time, which should be considered in prevention programs.

## Supporting information

S1 File Moderator models. Table A. Self-efficacy, family climate, and social support moderating the relationship between mental health problems and health-related quality of life in children and adolescents. Table B. Self-efficacy, family climate, and social support moderating the relationship between parental mental health problems and health-related quality of life in children and adolescents.
(DOCX)

S2 File Dataset. (XLS)
