# Supplementary material for: Risk and protective factors of health-related quality of life in children and adolescents: Results of the longitudinal BELLA study
Source: PLoS One. 2017 Dec 28;12(12):e0190363. doi: 10.1371/journal.pone.0190363 (PMC5746247; doi:10.1371/journal.pone.0190363)
Supplement: S1 File — Table A. Self-efficacy, family climate, and social support moderating the relationship between mental health problems and health-related quality of life in children and adolescents. Table B. Self-efficacy, family climate, and social support moderating the relationship between parental mental health problems and health-related quality of life in children and adolescents. (DOCX) [file pone.0190363.s001.docx]

**Supporting Information. S1 File. Moderator models.**

Manuscript title:

**Risk and protective factors of health-related quality of life in children and adolescents: results of the longitudinal BELLA study**

Journal:

PLOS One

Authors:

Christiane Otto^1*¶^, Anne-Catherine Haller^1¶^, Fionna Klasen^1^, Heike Hölling^2^, Monika Bullinger^3^, Ulrike Ravens-Sieberer^1^, on behalf of the BELLA study group^

^1^ Department of Child and Adolescent Psychiatry, Psychotherapy, and Psychosomatics, University Medical Center Hamburg-Eppendorf, Hamburg, Germany

^2^ Department of Epidemiology and Health Monitoring, Robert Koch-Institute, Berlin, Germany

^3^ Institute and Outpatients Clinic Medical Psychology, Center for Psychosocial Medicine, University Medical Center Hamburg-Eppendorf, Hamburg, Germany

*Corresponding author:

Email: c.otto@uke.de (CO)

^¶^These authors contributed equally to this work.

^Membership of the BELLA study group is provided in the Acknowledgments of the manuscript.

**S1A Table. Self-efficacy, family climate, and social support moderating the relationship between mental health problems and health-related quality of life in children and adolescents.**

|  | **Regression Model A1^1^**  **predicting initial HRQoL** | | | **Regression Model B1^2^**  **predicting change in HRQoL** | | |
| --- | --- | --- | --- | --- | --- | --- |
|  | ***b*** | **β** | ***p*** | ***b*** | **β** | ***p*** |
| Constant | 3.17 |  | <.001 | 0.05 |  | <.001 |
| Female | -0.10 | -.14 | <.001 | -0.01 | -.06 | .014 |
| Age (in years at baseline) | -0.03 | -.17 | <.001 | 0.00 | .03 | .441 |
| Age by gender | -0.01 | -.05 | .042 | 0.00 | .09 | .008 |
| Socioeconomic status (at baseline) | -0.01 | -.07 | <.001 | 0.00 | .03 | .184 |
| Migration background | -0.06 | -.05 | .014 | -0.01 | -.04 | .104 |
| **Risk factor** |  |  |  |  |  |  |
| Initial mental health problems (intercept) | -0.77 | -.36 | <.001 | 0.00 | .00 | .900 |
| Change in mental health problems (slope) |  |  |  | -0.27 | -.28 | <.001 |
| **Protective factors** |  |  |  |  |  |  |
| Initial self-efficacy (intercept) | 0.22 | .17 | <.001 | -0.01 | -.03 | .343 |
| Change in self-efficacy (slope) |  |  |  | 0.07 | .15 | <.001 |
| Initial family climate (intercept) | 0.15 | .16 | <.001 | -0.01 | -.05 | .101 |
| Change in family climate (slope) |  |  |  | 0.02 | .04 | .085 |
| Initial social support (intercept) | 0.14 | .17 | <.001 | 0.00 | .02 | .436 |
| Change in social support (slope) |  |  |  | 0.04 | .09 | .001 |
| **Interactions between risk and protective factors** |  |  |  |  |  |  |
| Initial mental health problems by initial self-efficacy | 0.06 | .01 | .709 | 0.01 | .01 | .765 |
| Change in mental health problems by change in self-efficacy |  |  |  | -0.06 | -.01 | .718 |
| Initial mental health problems by change in self-efficacy |  |  |  | -0.01 | -.00 | .901 |
| Change in mental health problems by initial self-efficacy |  |  |  | 0.04 | .01 | .690 |
| Initial mental health problems by initial family climate | 0.06 | 0.01 | .623 | -0.01 | -.01 | .701 |
| Change in mental health problems by change in family climate |  |  |  | -0.01 | -.00 | .939 |
| Initial mental health problems by change in family climate |  |  |  | -0.03 | -.01 | .623 |
| Change in mental health problems by initial family climate |  |  |  | 0.09 | .04 | .208 |
| Initial mental health problems by initial social support | -0.13 | -0.03 | .177 | 0.02 | .03 | .321 |
| Change in mental health problems by change in social support |  |  |  | -0.07 | -.01 | .748 |
| Initial mental health problems by change in social support |  |  |  | 0.01 | .00 | .922 |
| Change in mental health problems by initial social support |  |  |  | -0.11 | -.05 | .066 |

^1^ Linear regression Model A1 (*n* = 1,554); model fit: adjusted *R²* = .49; *F* = 123.62; ^2^ Linear regression Model B1 (*n* = 1,554); model fit: adjusted *R²* = .16; *F* = 12.57; HRQoL = health-related quality of life; *b* = unstandardized regression coefficient; β = standardized regression coefficient; for measures see main manuscript (Methods).

**S1B Table. Self-efficacy, family climate, and social support moderating the relationship between parental mental health problems and health-related quality of life in children and adolescents.**

|  | **Regression Model A2^1^**  **predicting initial HRQoL** | | | **Regression Model B2^2^**  **predicting change in HRQoL** | | |
| --- | --- | --- | --- | --- | --- | --- |
|  | ***b*** | **β** | ***p*** | ***b*** | **β** | ***p*** |
| Constant | 3.18 |  | <.001 | 0.05 |  | <.001 |
| Female | -0.13 | -.19 | <.001 | -0.01 | -.07 | .009 |
| Age (in years at baseline) | -0.03 | -.15 | <.001 | 0.00 | .02 | .532 |
| Age by gender | -0.02 | -.08 | .008 | 0.00 | .10 | .004 |
| Socioeconomic status (at baseline) | -0.00 | -.05 | .009 | 0.00 | .02 | .477 |
| Migration background | -0.07 | -.05 | .011 | -0.01 | -.04 | .115 |
| **Risk factor** |  |  |  |  |  |  |
| Initial parental mental health problems (intercept) | -0.11 | -.12 | <.001 | 0.00 | .02 | .586 |
| Change in parental mental health problems (slope) |  |  |  | -0.05 | -.04 | .112 |
| **Protective factors** |  |  |  |  |  |  |
| Initial self-efficacy (intercept) | 0.35 | .26 | <.001 | 0.00 | .01 | .630 |
| Change in self-efficacy (slope) |  |  |  | 0.10 | .21 | <.001 |
| Initial family climate (intercept) | 0.22 | .24 | <.001 | -0.01 | -.06 | .057 |
| Change in family climate (slope) |  |  |  | 0.03 | .07 | .014 |
| Initial social support (intercept) | 0.16 | .20 | <.001 | 0.00 | .03 | .305 |
| Change in social support (slope) |  |  |  | 0.04 | .09 | .001 |
| **Interactions between risk and protective factors** |  |  |  |  |  |  |
| Initial parental mental health problems by initial self-efficacy | 0.05 | .02 | .493 | 0.01 | .01 | .631 |
| Change in parental mental health problems by change in self-efficacy |  |  |  | -0.15 | -.02 | .515 |
| Initial parental mental health problems by change in self-efficacy |  |  |  | -0.03 | -.03 | .347 |
| Change in parental mental health problems by initial self-efficacy |  |  |  | 0.09 | .02 | .443 |
| Initial parental mental health problems by initial family climate | 0.02 | .01 | .698 | 0.01 | .05 | .165 |
| Change in parental mental health problems by change in family climate |  |  |  | 0.15 | .02 | .500 |
| Initial parental mental health problems by change in family climate |  |  |  | 0.03 | .03 | .299 |
| Change in parental mental health problems by initial family climate |  |  |  | 0.00 | .00 | .977 |
| Initial parental mental health problems by initial social support | 0.00 | .00 | .992 | -0.00 | -.02 | .626 |
| Change in parental mental health problems by change in social support |  |  |  | 0.06 | .01 | .804 |
| Initial parental mental health problems by change in social support |  |  |  | 0.05 | .04 | .144 |
| Change in parental mental health problems by initial social support |  |  |  | -0.11 | -.05 | .144 |

^1^ Linear regression Model A2 (*n* = 1,554); model fit: adjusted *R²* = .41; *F* = 89.57; ^2^ Linear regression Model B2 (*n* = 1,554); model fit: adjusted *R²* = .08; *F* = 6.66; HRQoL = health-related quality of life; *b* = unstandardized regression coefficient; β = standardized regression coefficient; for measures see main manuscript (Methods).
